# The new definition of early Health Technology Assessment: implications for incorporating environmental sustainability

**DOI:** 10.1017/S0266462325100330

**Published:** 2025-07-21

**Authors:** Melissa Pegg, Sarin K C, Abhirup Dutta Majumdar, Sabrina Grigolo, Janek Kapper, Matthew Hayden Gray Taylor

**Affiliations:** 1 York Health Economics Consortium (YHEC), University of York, United Kingdom; 2 Health Technology Assessment International (HTAi), Environmental Sustainability in Health Technology Assessment (ESHTA) Working Group, Edmonton, Alberta, Canada; 3 Health Intervention and Technology Assessment Foundation Program (HITAP), Nonthaburi, Thailand; 4 Advanced Analytics, PharmaQuant Insights Pvt Ltd, Kolkata, WB, India; 5 European Patients’ Academy on Therapeutic Innovation (EUPATI), EUPATI Foundation, Daltonlaan 600, 3584BK Utretcht, The Netherlands; 6 Estonian Inflammatory Bowel Disease Society, Ümera 11-172, Tallinn, Harjumaa, Estonia; 7 Counties Manukau Health, Te Whatu Ora, Auckland, New Zealand

**Keywords:** early health technology assessment, environmental sustainability, environmental aspects

## Abstract

**Objectives:**

The objective of this paper is to present the Environmental Sustainability in Health Technology Assessment (ESHTA) Working Group’s (WG’s) opinion on the definition and scope of early Health Technology Assessment (HTA) developed by a WG under HTA International. The aim is to provide suggestions on how early HTA can support the goals of enhancing environmental sustainability in healthcare.

**Methods:**

The HTAi ESHTA WG presents our opinion on the proposed definition and scope of early HTA. This includes a broad range of perspectives from stakeholder groups including patient experts, a policy maker, a statistician, HTA researchers and a healthcare professional, located across lower to higher resource settings and several jurisdictions. We suggest how early HTA can support the goals of enhancing environmental sustainability in healthcare.

**Results:**

HTA agencies play a crucial role in embedding sustainability into their evaluations and practices. Integrating environmental sustainability into HTA at three critical stages – product conceptualization, reimbursement decisions, and point of care – can optimize resource use and reduce environmental impacts. Developing sustainability metrics, defining environmental impact categories, and identifying suitable methods for assessing health technologies are essential steps. Early engagement is also vital for optimizing trade-offs and increasing acceptance by diverse stakeholders.

**Conclusions:**

Incorporating environmental sustainability into early HTA can enhance the likelihood of regulatory approval and reimbursement, ultimately benefiting patients and healthcare systems. By integrating sustainability considerations at the design stage, the potential for environmental impact reduction is maximized. Future efforts should focus on developing comprehensive guidelines and methods, ensuring collaboration between early HTA and ESHTA WGs.

## Introduction

A consensus-based definition of early Health Technology Assessment (HTA) has been developed by a working group (WG) under HTA International (HTAi). The WG defines early HTA as “a health technology assessment conducted to inform decisions about subsequent development, research, and/or investment by explicitly evaluating the potential value of a conceptual or actual health technology.” Furthermore, the WG states that early HTA expanded the notion of value to include “environmental aspects.” In this perspective, the HTAi Environmental Sustainability in Health Technology Assessment (ESHTA) WG presents our opinion on the proposed definition and scope of early HTA. We suggest how early HTA can support the goals of enhancing environmental sustainability in healthcare.

## Environmental sustainability in HTA (ESHTA)

Global society currently encounters unprecedented challenges, including climate change, resource depletion, chemical contamination, and increasing social problems (1). Moreover, anthropogenic effects across all environmental domains, including air, water, and soil pollution, are threatening planetary and human health (2). There is growing research related to the healthcare sector’s contribution to environmental destabilization (3). This has prompted the global healthcare community to commit to reducing its environmental impact (3). As such, health systems globally are developing national mitigation plans to achieve net zero (4;5). However, these plans are primarily focused on the system level, for example, transitioning to renewable energy and technologies that support more sustainable healthcare facilities. Corroborate this with evidence showing that 61 percent (18.9 million tons) of carbon emissions in the United Kingdom National Health Service Carbon Footprint Plus occur in the supply chain, contributed by 80,000 suppliers (6).

The opportunity to enhance environmental sustainability both at the product level and across care pathways remains at large in HTA (7). Broader challenges, such as a paucity of government environmental targets and linked health policy targets across most countries, further constrain the incorporation of environmental sustainability in HTA (8). More recently, coronavirus disease 2019 has put further strain on healthcare sustainable development, meaning that most healthcare systems are predominantly agnostic in incorporating environmental sustainability (9). However, there is an increasing call by the HTA community to respond to global climate targets and sustainable development goals, including the additional dimension of environmental aspects (10).

A recent scoping review and bibliometric analysis (11) reported a limited number of methods to evaluate the environmental sustainability of health technologies, the majority of which are concentrated in the United States, Australia, Canada, and the United Kingdom. Also recognizing the current state of play, the International Agency for Health Technology Assessment recently published a white paper to advance the discussion of the potential readiness and role of HTA agencies to integrate environmental assessment into their evaluations (12). The issue is burdened by the fact that there is neither consensus among the HTA community on the definition and scope of ESHTA nor on the appropriate methods for its evaluation (13–15). Nonetheless, to address this gap, a WG under HTAi was formed in May 2024, that is, ESHTA, with three main objectives as follows: (i) to develop a consensus-based definition of ESHTA; (ii) to define the criteria/scope of ESHTA; and (iii) to assess the fitness of existing methods to incorporate ESHTA.

## Early HTA and ESHTA

The question from an ESHTA perspective is, “How can early HTA provide systematic guidance that optimizes sustainability in technology development and early product development?.” Approximately 80 percent of the total environmental impact of a product is determined at the research and development (R&D) stage (16). Therefore, it is crucial that health technology developers integrate sustainability considerations into their business processes. Novel and innovative technologies should be encouraged to support sustainability and mitigate environmental harm across the life cycle of products. This will help avoid the shortage and depletion of natural resources, reduce air, water, and soil pollution, minimize environmental and biodiversity degradation, and preserve the biosphere for a sustainable future of human society (17;18).

The ESHTA WG welcomes the proposed definition of early HTA, as it opens the door for incorporating ESHTA. At the product level, decisions about environmental sustainability can occur at three crucial stages. First, before the products have been conceptualized or developed. Second, while making reimbursement decisions. Finally, while using those products at the point of care (including downstream environmental considerations at the stage of disposal, end of life, and return to nature). Specifically, we propose that the first two stages provide the greatest opportunity to reduce the environmental impacts of prospective health technologies. For example, a focus on developing innovative technologies that spearhead sustainability will also optimize resource use across care pathways and reduce downstream environmental impacts such as waste management. This aligns with a growing demand by the regulators for industry to transparently and adequately disclose environmental data and demonstrate reductions in the environmental life cycle of their products, an area that Jeswani and Azapagic (19) can help address. Consecutive application of these techniques will help to fill the data and evidence void that currently exists (15;20;21) while developing product sustainability dimensions, inter- and intra-organizational actors, decision-making support tools, and life cycle information flows (22;23).

Although not formally included in reference cases to date, HTA agencies like the Scottish Health Technologies Group, England’s National Institute for Health and Care Excellence, Canada’s Drug Agency, Zorginstituut Netherlands, and the Health Intervention and Technology Assessment Program Foundation (HITAP), Thailand are now beginning to consider incorporating environmental sustainability into their assessments (24;25). Therefore, provided the definition of “value” of health technologies is extended to environmental sustainability, as alluded to by the early HTA WG, future innovators will benefit from ensuring their products are more environmentally sustainable while benefiting patients and overall healthcare sustainability. It is proposed that this will maximize the likelihood of getting regulatory approval and reimbursement.

Environmental sustainability considerations have not influenced the development and reimbursement of many of the health technologies we use today. Despite our belief that prevention is better than cure (i.e., ESHTA in the first two stages of early HTA is most preferred), all hope is not lost for the technologies that are already reimbursed. Existing sustainability frameworks, such as the Sustainable Quality Improvement (SusQI) from the Centre for Sustainable Healthcare, focus on holistic health technology solutions that together fulfill health, environmental, financial, and social goals (26). This includes identifying and reducing low-value care. Although there are obvious co-benefits of incorporating ESHTA in terms of health benefits, financial savings, and environmental protection, potential relative trade-offs and opportunity costs may arise, underlining the importance of transdisciplinary collaboration and standardization of methods (27).

## Early HTA stakeholder engagement and ESHTA

The ESHTA WG proposes that knowledge in this topic will prove useful to the early HTA community and innovators, who can optimize any climate-health-economic trade-offs that may arise during the product conceptualization stage and increase their likelihood of being accepted by diverse stakeholders. Importantly, early HTA draws on a body of complementary methods. For example, patient interviews, expert (stakeholder) elicitation, statistical analyses, and health economic modeling are used to assess the need for the commercial feasibility of a health technology using target product profiles (TPPs). Current TPPs rarely focus on defining aspirational environmental impact goals and are typically restricted to containing data on efficacy, safety, dosing, and other technology characteristics. However, if considered, this approach is likely advantageous for the technology developer because the entire life cycle and associated methods can be increasingly integrated during the setting up of good manufacturing practices. For example, when transitioning from Stage 2 to Stage 3 of product development. Furthermore, we are aware that the industry appreciates both the benefits and opportunities of considering environmental components early in development. In addition, many innovators are committed to delivering transformational therapies while minimizing the environmental impact of their operations and products. However, it will take time to systemically embed sustainability into drug development/early HTA, owing to several factors, including the complexity of the R&D process and the strict regulatory environment within which industry operates. Thus, the feasibility for innovators to achieve sustainability goals is made attainable, provided that basic conditions are met. For example, clarity and prospective measures to incorporate environmental sustainability in early HTA include the following:

1. If/how incorporating environmental sustainability would be rewarded by payers/healthcare systems.
2. Optimal routes of administration and posology.
3. Environmental impact of drug interventions along healthcare pathway(s).
4. Knowledge of expected supply chain logistics.
5. Incorporating environmental sustainability aspects during the device development phase.

Early dialogue with the patient and caregivers as “value holders” rather than stakeholders is critical to mitigate the mismatch between the patients’ needs and research findings, including early HTA. Thus, the patient’s voice and needs should be included at the earliest stages of HTA (early HTA). A conceptual study by Pegg et al. (28) demonstrates it is possible to elicit the public’s view about trade-offs between direct health outcomes and the environment. Imperatively, the study highlights that environmental policies that solely focus on carbon emissions are likely to undervalue the public’s preference for the environment and may be unsustainable in the long term than the inclusion of more holistic environmental outcomes. Accounting for public preferences, decision-making can be optimized to support greater societal benefit (29). We recommend that countries undertake similar studies to establish acceptable relative trade-offs between health outcomes and environmental goals, although there are undisputable limitations to this approach, including the unavoidable consideration that harm to the environment can directly lead to human ill health, loss of human life, and a reduction in quality of life (30).

To begin to address this issue, Hensher (31) proposes that the environmental impacts of health technologies are included in health economics by costing negative externalities using shadow pricing. Therefore, developing the “environmental cost” of more sustainable healthcare requires patient and public involvement and engagement. Methods may include participating in focus groups, providing feedback in pilot projects, and joining a planning-phase WG (32). Furthermore, considering the patient’s perspective, we propose the following methodological considerations will help support the development of more sustainable health technologies during early HTA:

1. How might information be gathered from patients about their day-to-day lives?
2. What methods could be used to gauge the preferences of patients and caregivers?
3. What (if any) additional burdens might be created, for example, are patients willing to accept changes to their pathway/process or the use of a digital technology, if it helps reduce waste and carbon emissions?
4. How and when might patients be included across the early HTA techniques, discussions, and deliberations, what types of questions are asked, and how are patients’ views captured in decision-making?

Accordingly, ESHTA recommendations will be useful to early HTA researchers/innovators because these will point to data and evidence generation needs. Sustainability metrics important to stakeholders include acting on climate change to improve health and support transforming health systems “to be climate-resilient, low-carbon, sustainable, and equitable” (33). Thus, mandated healthcare systems typically measure their environmental impact by reporting changes to GHG emissions, energy use, and waste stream volumes (2;6). We propose that the areas to be defined include:

1. The goal and scope of ESHTA, including boundary considerations of health technology environmental assessment, with specific consideration to the current healthcare policy landscape.
2. Broad environmental outcomes, within the ESHTA boundary, that is, specific environmental impact categories (e.g., biodiversity, fossil fuel use, and waste volumes) are defined that are crucial to evaluate before reimbursement decisions are made.
3. Identification of appropriate methods and/or frameworks to translate data into useful evidence and to measure the environmental sustainability of products.
4. Methods and reporting checklists need to be complementary and coherent for product developers and health technology decision-making entities.

## Conclusion

Both ESHTA and early HTA aim to redefine the value of health technologies, going beyond health and economic benefits. We endorse the proposed definition of early HTA, as it allows environmental sustainability to be incorporated at the design stage, where it can be maximized. Notwithstanding, striving for a realistic balance between the opportunities and challenges of incorporating environmental sustainability in early HTA/ early product development is necessary. Future work of the early HTA WG includes the development of methodological and reporting guidelines. We recommend that the early HTA WG coordinate with the ESHTA WG for both work packages, such that crucial ESHTA items can be integrated into early HTA methods and reporting checklists, given that the regulators and HTA agencies will require these soon.
